# A preliminary study on the effects of E-Z Mixin® and EquiPlus® extenders supplemented with Edible Bird’s Nest on the quality of chilled Arabian stallion semen

**DOI:** 10.1590/1984-3143-AR2020-0027

**Published:** 2021-06-21

**Authors:** Khalid Al-Khaldi, Nurhusien Yimer, Samir Al-Bulushi, Abd Wahid Haron, Mark Hiew, Abdul Salam Babji

**Affiliations:** 1 Department of Veterinary Clinical Studies, Faculty of Veterinary Medicine, Universiti Putra Malaysia, Selangor, Malaysia; 2 Equine Breeding Center, Veterinary Department, Royal Oman Police, Mounted Police Division, Muscat, Sultanate of Oman; 3 Laboratories and Animal Research Centre, Directorate General of Veterinary Services, Royal Court Affairs, Muscat, Sultanate of Oman; 4 School of Chemical Sciences and Food Technology, Faculty of Science and Technology, Universiti Kebangsaan Malaysia, Bangi, Selangor, Malaysia

**Keywords:** Arabian stallion, EBN, chilled semen, EquiPlus®, E-Z Mixin®

## Abstract

The aim of this study was to evaluate the effects of adding different concentrations of edible bird’s nest (EBN) which is secreted by swiftlet birds (*Aerodramus fuciphagus*), into EquiPlus® and E-Z Mixin® extenders on the quality of chilled Arabian stallion semen at various storage times (0, 24 and 48 h). Ten ejaculates were collected from five stallions, and diluted using the two extenders containing 0% (control), 0.12%, 0.24% and 0.24% of EBN + seminal plasma (SP). All the diluted semen samples were then cooled and stored at 5 °C, and examined at 0, 24 and 48 h. Sperm kinetic parameters were assessed using computer assisted sperm analysis (CASA) and viability were assessed using Hoechst33342/PI stain. In both extenders, total motility (TM) and progressive motility (PM) were significantly higher at 0.12% and 0.24% compared to 0.24% + SP at 24 and 48 h. At 0.12%, E-Z mixin® treated semen had significantly higher TM and PM than EquiPlus^®^ at 24 and 48 h. At 0.12% and 0.24%, average path velocity (VAP), straight-line velocity (VSL) and curvilinear velocity (VCL) were significantly higher in E-Z mixin® treated semen compared to EquiPlus^®^ at 24 and 48 h. Comparisons between the two extender types at different concentrations of EBN showed no significant difference in lateral head amplitude (ALH), linearity (LIN), straightness (STR), beat cross frequency (BCF) and viability, irrespective of the storage time. The percentage of viable was significantly higher in E-Z mixin® than EquiPlus® at 0 and 48 h in control and 0.12%. Supplementation of the E-Z mixin^®^ extender with 0.12% and 0.24% EBN concentrations in the absence of SP provided better CASA parameters such as TM, PM, VAP, VSL, and VCL at 24 and 48 h storage time. In conclusion, the results of this study indicated that chilled semen from Arabian stallion that was extended using E-Z mixin® and supplemented with 0.12% and 0.24% EBN concentrations performed better and yielded superior results in sperm kinetic parameters and % viable compared to EquiPlus® at 24 and 48 h storage time.

## Introduction

Edible bird’s nest (EBN) has recently become a major focus in elucidating its potential role as a natural antioxidant (Ghassem et al., 2017). EBN is a product of the salivary secretions of swiftlet birds (*Aerodramus fuciphagus*) found in Malaysia and other South East Asia countries. EBN is a well-known valued product among the Chinese and has been consumed for centuries due its medicinal and nutritional properties (Wong, 2013), which include anti-aging (Hu et al., 2016), anti-oxidative, anti-inflammatory (Albishtue et al., 2019; Yida et al., 2015), as well as maintenance of sperm morphology during preservation (Yusop et al., 2016).

Chilled and liquid preservation of stallion semen is a good alternative for semen storage, as cryopreserved sperm are often exposed to damaging effects associated with the preservation technique. However, less than 50% of stallions produce semen that is suitable for freezing or cryopreservation and sperm freezability varies between breeds of horses (Vidament et al., 2001; Alvarenga et al., 2003; Al-Essawe et al., 2018a; Alamaary et al., 2019). Prior to insemination of mares, stallion semen is frequently subjected to cooled storage, but the procedure is associated with lower pregnancy rates compared to the use of fresh semen by natural mating or artificial insemination (Squires et al., 2004). Such low fertility rates were influenced by reductions in sperm motility, chromatin quality, plasma membrane intactness and high reactive oxygen species (Hernández-Avilés et al., 2020).

Seminal plasma is a compound mix of proteins and other substances. The effect of SP on the cryo-survival of spermatozoa cells is quietly debatable: some researchers claim positive effects, whereas others have seen adverse effects (Al-Essawe et al., 2018a).

EBN is a natural product composed of macro-nutrients such as protein and carbohydrates which are considered to be the major components (Ma and Liu, 2012; Marcone 2005). In addition, EBN contains other components including fat (< 2%) and trace amounts of minerals such as sodium and magnesium. The glycoprotein component of EBN consists of bioactive compounds such as sialic acid that contributes to increase metabolism and physiological functions in mammals (Yagi et al., 2008; Ahmad et al., 2019) and continues to receive more attention based on its anti-oxidation and other health-related benefits (Hou et al., 2015; Ghassem et al., 2017). Owing to its antioxidant property, EBN was reported to have positive effect in maintaining bull sperm morphology during cryopreservation (Yusop et al., 2016). Nevertheless, the benefits of EBN in preserving semen quality in stallion has not been investigated. Since oxidative damage and osmotic shock are amongst the main events leading to sperm damage during thawing and cooling process, the supplementation with EBN could ameliorate some of these alterations; thus, preserving semen quality. The present study was designed to evaluate the effect of different concentrations of EBN added into EquiPlus® and E-Z Mixin® extenders on the quality of chilled Arabian stallion semen.

## Materials and methods

### Animals

Five healthy Arabian stallions aged between 6 - 13 years old with weight ranging from 400 – 500 kg were used for the study. Each stallion was housed in 20 m^2^ area stable at the Mounted Police Division in Royal Oman Police area, Muscat, Oman. The Stallions were all provided with food and water ad libitum. The daily diet was made up of Katambora dry grass, oats, and concentrated ration cubes of HAVENS® basis-sport contain (carbohydrates 51%, crude protein 12.6%, crude fat 3.4%, calcium 10.4g and phosphorus 4.7). The experiment was conducted during the breeding season in Oman from January to May 2019. The ethical approval for all the procedures was obtained from the Animal Welfare Committee at Animal Health Research Center (AHRC), Directorate General of Agricultural & Livestock Research in Ministry of Agricultural and Fisheries, Sultanate of Oman, dated 12.3.2019 No. 54.

### Preparation of EBN and extenders

EBN hydrolysate obtained from Universiti Kebangsaan Malaysia (UKM) and maintained at 25-30 °C were used in this experiment. The two commercial extenders used in this experiment were EquiPlus® with main compositions of dry milk and gentamycin (Minitube, Germany) and E-Z Mixin® which is composed of glucose, non-fat dry milk solids and amikacin sulfate (Colorado State University), whereas the EBN was of house nest swiftlet (*Aerodramus fuciphagus*) origin in a hydrolyzed form. Initially, two stock solutions of EBN were prepared. Stock (A) hydrolyzed EBN solution was prepared by adding 0.24 g (240 mg) of hydrolyzed EBN into a 10 ml phosphate-buffered saline (PBS) (Sigma) to obtain hydrolyzed EBN solution aliquots of 2.4% concentration. Meanwhile, stock (B) of a hydrolyzed EBN solution was prepared by adding 0.12 g (120 mg) to a 10 ml PBS to obtain a hydrolyzed EBN solution aliquots of 1.2% concentration. The 10 ml stock (A) and (B) were stored in a freezer (-20 °C) in smaller fractions of 40 μl Eppendorf tubes before use. The aliquots were introduced into a water bath at 37 °C for 3 min before they were added to the extenders. A 40 μl of the hydrolyzed EBN (24 μg/μl) from stock A solution was added into 3960 μl of both extenders (EquiPlus® or E-Z mixin®) to make the 0.24% hydrolyzed EBN treatment. The same quantity of EBN from stock B solution (12 μg/μl) was added into in 3960 μl of both extenders (EquiPlus® or E-Z mixin®) to make the 0.12% of hydrolyzed EBN treatment.

### Experimental design

Ejaculates were divided equally and allocated into two main groups (E-Z Mixin® and EquiPlus®). Each sample at this stage were diluted with semen extenders: (1) E-Z Mixin® and (2) EquiPlus®. The dilutions of the samples were performed in two stages. Divided ejaculates were extended at ratio 1:1 with one of the treated diluents group (EquiPlus® or EZ- Mixin®) and centrifuged with Maxifreeze® (IMV, France) anti-shock cushion at 600 x g for 10 min (Dell' aqua et al., 2001). The supernatant was filtered, and the sperm pellet suspended into a final concentration of 50 million sperm/ml with one group of the commercial chilled treated semen extender (EquiPlus® or EZ-Mixin®) before they were transferred into cold cabinet (mini-tube, German). Thereafter, each group were divided into four sub-groups (A, B, C, D) corresponding to the addition of 4 ml of various concentrations of EBN (control, 0.12%, 0.24%, 0.24% + SP). In both groups, the control group represented the negative control (no addition of EBN) and 0.24% + SP represented group containing seminal plasma. All the diluted semen samples were then cooled and stored at 5 °C for 24 and 48 h.

### Semen collection and processing

A total of ten ejaculates were collected from five stallions (2 ejaculates/stallion). The semen was collected using a Hannover model artificial vagina (AV) (Mini-tube of America, Verona, WT), which was lubricated using a non-spermicidal lubricant (Mini-tube, Germany) and pre-warmed at 48 °C. A phantom was used for the collection in the presence of an estrous mare to stimulate the stallion. The ejaculate was separated from the gel using an inline filter fitted into the AV (Mini-tube, Germany).

Semen volume (mL) was determined using a graduated cylinder. A drop of gel-free semen was placed on a warm (37 °C) microscope slide and viewed under low power. As described by Dowsett and Knott (1996), density and mass activity was a visual estimate of sperm concentration and overall activity, respectively. Sperm concentration was determined using SDM1-Photometer (Mini-tube, German). As shown in subsequent section, sperm kinetics, as well as plasma and viability were assessed at this time. Only ejaculates with sperm motility greater than 60% and concentrations of 250 x 10^6^ sperm cells/mL were recruited for the experiment.

### Evaluation of sperm kinetics

Parameters of sperm kinetics were assessed using the Computer-Assisted Sperm Analyzer (CASA; CEROS, Version12, Hamilton Thorne Biosciences, USA). A sample (3 µL) from each tube was put placed on a pre-warmed Leja slide (standard count 4 chamber slide, 20 micron, Leja, Nieuw-Vennep, The Netherlands), semen was evaluated based on six digitalized images from different fields through a 20 × negative-phase contrast objective at 37 °C. The parameters recorded included motility variables (total and progressive motility; %), curvilinear velocity (VCL; µm/s), straightness (VSL; µm/s), straightness index (STR; %), average path velocity (VAP; µm/s), linearity (LIN; %), beat cross frequency (BCF; Hz), and amplitude of lateral head deviation (ALH; µm). Standard CASA setting was used to analyze the semen (Hamilton Thorne); settings and cut-off points used for CASA are presented in Table 1.

**Table 1:** Settings of the Hamilton Thorne CEROS animal software (Version 12) used to assess stallion sperm kinematics.

**Variable**	**Setting**
Frame rate	60 Hz
Number of frames required	45
Minimum contrast	55
Minimum cell size (pixels)	6
Progressive path velocity cut-off	30 μm/s
Progressive straightness	60%
Slow average path velocity cutoff	10 μm/s
Slow straight line velocity cutoff	5 μm/s
Static average path velocity cutoff	4 μm/s
Static average line velocity cutoff	1 μm/s
Non-motile head size (pixels)	0.5 - 4.8
Non-motile head intensity	0.25 - 1.8
Non-motile head intensity	1.87 x
Video frequency	60
Illumination intensity	2300
Temperature	37 °C

### Sperm viability - vital test

The viability of the sperm was assessed with Hoechst33342/PI stain (Mini-tube, Germany); a fluorescent stain for determining membrane integrity of semen samples. The staining was used to distinguish semen cells with intact and damaged membrane. The staining was analyzed with the module “Membrane Integrity” (Viability) of AndroVision® (Mini-tube, Germany). Initially the semen sample (50 µL; 200 million spermatozoa/ml concentration) was warmed at 37 °C. Then 1.5 µL of stain was added to 50 µL of the semen sample and mixed before incubating for 15 min. Thereafter, a drop of the mixed semen sample was placed on a microscope slide, covered with a cover slip and evaluated by counting 500 sperm using AndroVision® Florescent microscope – Neofluar objective (20x) at 37 °C.

### Statistical analysis

All the data were analyzed using the SPSS statistical software (Version 24, IBM, USA). All the CASA parameters and morphological properties were analyzed using repeated measures analysis of variance (ANOVA) under the General linear model. The fixed factors were the two semen extenders. The CASA parameters were checked for normality using the Shapiro Wilk test. Time (0, 24 and 48 h), extender (EquiPlus® and E-Z mixin®), EBN concentration (control, 0.12%, 0.24%, 0.24% + P) were considered as fixed effect in the model. *P < 0.05* was considered for statistically significance.

## Results

**Figure 1:**
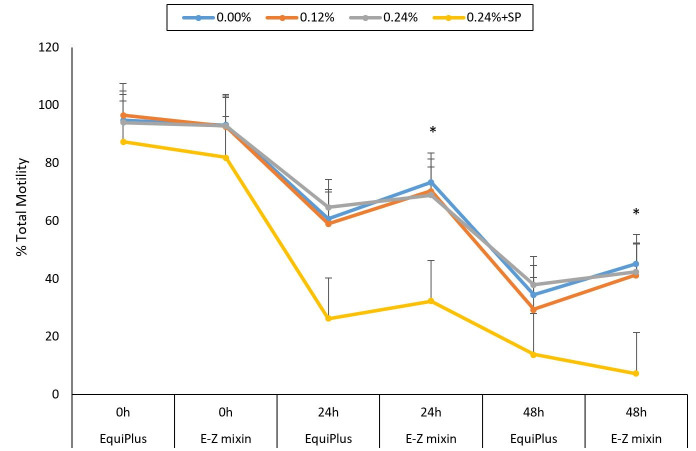
Percentage of Total Motility (TM) extended with E-Z mixin® and EquiPlus® extenders supplemented with 0.12% and 0.24% of EBN with/without SP removal and checked at 0, 24 and 48 h of storage time. Note: Note: Comparisons is between the two extenders at each time points (0, 24, and 48 h) for each concentration of EBN (control, 0.12%, 0.24% and 0.24 +SP). Extenders with (*) on various points on the curve are significantly different.

**Figure 2:**
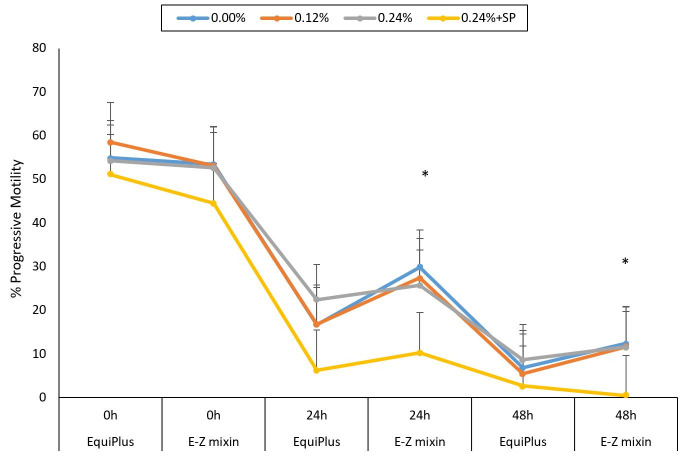
Percentage of Progressive Motility (PM) extended with E-Z mixin® and EquiPlus® extenders supplemented with 0.12% and 0.24% of EBN with/without SP removal and checked at 0, 24 and 48 h of storage time. Note: Comparisons is between the two extenders at each time points (0, 24, and 48 h) for each concentration of EBN (control, 0.12%, 0.24% and 0.24 +SP). Extenders with (*) on various points on the curve are significantly different.

**Figure 3:**
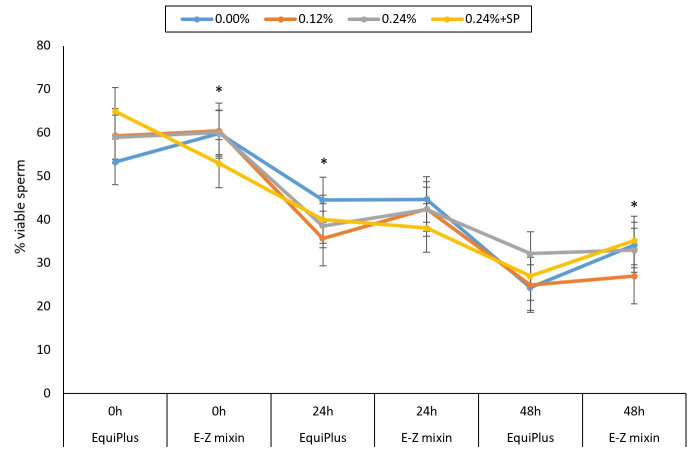
Percentage of viable sperm in samples extended with E-Z mixin® and EquiPlus® and supplemented with 0.0% (control), 0.12%, 0.24% of EBN with/without SP removal and checked at 0, 24 and 48 h of storage time. Note: Comparisons is between the two extenders at each time points (0, 24, and 48 h) for each concentration of EBN (control, 0.12%, 0.24% and 0.24 +SP). Extenders with (*) on various points on the curve are significantly different.

Table 2 shows the effects of supplementing the two extenders (E-Z mixin® and EquiPlus®) with different concentrations of EBN on sperm kinetic parameters at 0, 24 and 48 h, excluding TM, PM and percentage viable sperm (i.e. shown in Figure 1, 2 and 3). For EquiPlus®, at 0 h, there was no significant difference in sperm TM between different concentrations of EBN. At 24 and 48 h, sperm TM was not significantly different between control, 0.12% and 0.24%, but 0.24% had higher TM (*P < 0.05*) compared to 0.24% + SP. The results for E-Z mixin® at different concentrations of EBN for each time frame were similar to that of EquiPlus® (Figure 1). Based on the comparison of different time frame for each EBN concentration, the TM for EquiPlus® and E-Z mixin® in control decreased significantly (*P < 0.05*) from 0 to 48 h. Table 3 shows the comparison between the two diluents for each EBN concentration and storage time. For semen supplemented with 0.12% EBN, E-Z mixin® had higher *(P < 0.05)* TM than EquiPlus® at 24 and 48 h.

**Table 2:** Comparison of sperm kinetics between the different EBN concentrations (0.0%, 0.12%, 0.24% and 0.24% + SP) at 0, 24 and 48 h storage time in EquiPlus® and E-Z mixin® extenders.

**Variable**	**Time (h)**	**EquiPlus®**				**E-Z mixin®**			
		**0.0%**	**0.12%**	**0.24%**	**0.24% + SP**	**0.0%**	**0.12%**	**0.24%**	**0.24% + SP**
Path Velocity (VAP, µm/s)	0	168.4 ±17.5^A,a^	168.5 ±14.6 ^A,a^	158.7 ±4.2 ^A,a^	149.8 ±14.5 ^A,a^	152.0 ±17.5 ^A,a^	152.8 ±12.6 ^A,a^	137.1 ±5.1 ^A,a^	149.6 ±11.7 ^A,a^
	24	74. ±7.1 ^A,b^	75.4 ±11.4 ^A,b^	81.4 ±19.8 ^A,b^	57.1 ±10.5 ^B,b^	111.5 ±23.2 ^A,b^	108.1 ±12.4 ^A,b^	106.3 ±17.8 ^A,b^	73.5 ±5.2 ^B,b^
	48	64.7 ±4.4 ^A,b^	58.6 ±4.2 ^A,b^	60.6 ±7.5 ^A,b^	50.3 ±10.4 ^A,b^	88.8 ±13.5 ^A,c^	84.1 ±11.9 ^A,c^	84.1 ±11.5 ^A,c^	45.1 ±3.1 ^B,c^
Progressive velocity (VSL, µm/s)	0	130.2 ±18.3 ^A,a^	138.7 ±12.6 ^A,a^	129.5±19.7 ^A,a^	126.7±17.1 ^A,a^	125.4± 7.8 ^A,a^	125.8±12.8 ^A,a^	125.9±12.8 ^A,a^	129.3±14.7 ^A,a^
	24	53.6 ±2.4 ^A,b^	52.3 ±11.5 ^A,b^	59.1 ±8.3 ^A,b^	37.7±7.1 ^B,b^	86.2±2.4 ^A,b^	73.9±3.7 ^A,b^	80.4±7.9 ^A,b^	54.2±26.6 ^A,b^
	48	40.5 ±6.3 ^A,b^	38.4 ±2.5 ^A,b^	38.8 ±6.5 ^A,b^	32.7±8.5 ^B,b^	63.6±11.2 ^A,b^	62.4±8.3 ^A,b^	60.6±10.3 ^A,c^	25.2±4.5 ^A,c^
Curvilinear velocity (VCL, µm/s)	0	251.5±41.8 ^A,a^	277.5±23.7 ^A,a^	250.4±22.3 ^A,a^	245.4±23.3 ^A,a^	252.4±21.3 ^A,a^	252.8±12.6 ^A,a^	244.1±30.5 ^A,a^	220.1±27.3 ^A,a^
	24	131.7±11.6 ^A,b^	132.6±20.5 ^A,b^	141.4±14.1 ^A,b^	102.2±22.7 ^B,b^	192.3±48.6 ^A,b^	194.5±24.1 ^A,b^	191.1±23.3 ^A,b^	143.7±27.1 ^B,b^
	48	110.5±13.2 ^A,c^	107.4±6.3 ^A,c^	108.8±12.3 ^A,c^	84.0±18.2 ^A,c^	149.6±29.2 ^A,c^	158.1±25.4 ^A,c^	155.9±21.6 ^A,c^	82.2±13.6 ^B,c^
Lateral head amplitude (ALH, µm)	0	8.8±0.7 ^A,a^	9.1±0.9 ^A,a^	9.0±0.7 ^A,a^	8.3±0.8 ^A,a^	8.5±0.9 ^A,a^	8.5±0.4 ^A,a^	8.8±0.8 ^A,a^	8.0±0.8 ^A,a^
	24	6.1±0.6 ^A,a^	6.3±0.8 ^A,a^	6.3±1.3 ^A,a^	4.5±1.6 ^A,a^	8.5±1.6 ^A,a^	8.4±0.8 ^A,a^	8.5±0.9 ^A,a^	7.2±1.7 ^A,a^
	48	6.1±0.9 ^A,a^	5.7±0.4 ^A,a^	5.3±0.3 ^A,a^	3.3±1.9 ^A,a^	7.9±1.3 ^A,a^	8.4±1.4 ^A,a^	7.6±0.8 ^A,a^	4.0±4.3 ^A,a^
Beat cross frequency (BCF, Hz)	0	17.7±4.9 ^A,a^	17.6±3.3 ^A,a^	18.2±5.1 ^A,a^	23.6±5.6 ^A,a^	19.1±4.2 ^A,a^	19.2±5.6 ^A,a^	18.3±3.9 ^A,a^	21.7±2.6 ^A,a^
	24	14.3±2.7 ^A,a^	13.1±2.7 ^A,a^	13.7±2.5 ^A,a^	12.5±2.8 ^A,a^	18.1±2.7 ^A,a^	18.5±1.5 ^A,a^	17.1±3.8 ^A,a^	29.5±3.4 ^A,a^
	48	14.8±1.9 ^A,a^	15.1±4.0 ^A,a^	14.5±4.9 ^A,a^	10.9±5.4 ^A,a^	16.4±1.6 ^A,a^	18.8±3.2 ^A,a^	16.3±2.5 ^A,a^	17.8±9.1 ^A,a^
Straightness (STR, %)	0	77.6±4.3 ^A,a^	78.6±3.1 ^A,a^	77.3±3.1 ^A,a^	80.4±4.8 ^A,a^	77.6±4.3 ^A,a^	77.7±2.7 ^A,a^	77.7±3.1 ^A,a^	83.0±8.0 ^A,a^
	24	67.1±5.7 ^A,a^	64.3±4.6 ^A,a^	67.1±5.0 ^A,a^	62.9±6.8 ^A,a^	71.4±2.4 ^A,a^	72.7±1.7 ^A,a^	70.4±4.7 ^A,a^	63.0±8.5 ^A,a^
	48	62.7±6.1 ^A,a^	63.7±5.0 ^A,a^	63.7±3.4 ^A,a^	64.3±10.4 ^A,a^	68.1±3.4 ^A,a^	69.2±3.1 ^A,a^	68.7±3.8 ^A,a^	57.5±14.3 ^A,a^
Linearity (LIN, %)	0	47.1±3.3 ^A,a^	48.3±2.3 ^A,a^	47.0±1.64 ^A,a^	50.4±4.6 ^A,a^	47.4±4.4 ^A,a^	48.3±2.3 ^A,a^	47.6±3.6 ^A,a^	48.5±6.6 ^A,a^
	24	40.1±3.9 ^A,a^	38.7±2.7 ^A,a^	40.6±2.9 ^A,a^	40.0±7.1 ^A,a^	40.9±0.9 ^A,a^	38.7±2.7 ^A,a^	39.9±2.0 ^A,a^	37.0±4.3 ^A,a^
	48	38.0±3.1 ^A,a^	39.0±5.2 ^A,a^	38.5±2.6 ^A,a^	43.6±8.6 ^A,a^	39.1±2.3 ^A,a^	39.0±5.2 ^A,a^	39.3±2.9 ^A,a^	35.2±9.9 ^A,a^

Note: For each extender, lower-case superscripts were used to compare the means of sperm kinetic parameters at various storage time (0, 24 and 48 h) across the column for each EBN concentration. Upper-case superscripts were used for comparisons between the two semen extenders at specific storage time for different EBN concentration. Variables (mean ± SE) with different upper-case or lower-case superscripts indicate significant difference (*P < 0.05*).

**Table 3:** Comparison of sperm kinetics between EquiPlus® and E-Z mixin® extenders at each concentration of EBN at 0, 24 and 48 h storage time.

**Variable**	**Time (h)**	**EBN concentrations**							
		**0.0%**		**0.12%**		**0.24%**		**0.24%+SP**	
		**EquiPlus®**	**E-Z mixin®**	**EquiPlus®**	**E-Z Mixin®**	**EquiPlus®**	**E-Z Mixin®**	**EquiPlus®**	**E-Z Mixin®**
Total motility (%)	0	94.8±4.5 ^A^	93.1 ±6 .2 ^A^	96.6±2.7 ^A^	92.7 ±7 .1 ^A^	94.0 ±3.5 ^A^	92.9 ±5 .7 ^A^	87.4 ±9.4^A^	82.0 ±13.4 ^A^
	24	60.8±2.6 ^A,^	73.4 ±1.6 ^A^	59.1±2.2 ^A^	70.4 ±1.5 ^B^	64.7 ±2.0 ^A^	69.0 ±1.7 ^B^	26.2 ±2.1^A^	32.3 ±2.8 ^A^
	48	34.5±2.9 ^A^	45.2 ±2.2 ^A^	29.5±2.8 ^A^	41.4 ±2.9 ^B^	38.0 ±2.9 ^A^	42.4 ±2.5 ^B^	13.9 ±19.3^A^	7.3 ±3.1 ^A^
Progressive motility (%)	0	54.9±7.2 ^A^	53.4 ±7.9 ^A^	58.5 ±6.4 ^A^	53.1 ±3.9 ^A^	54.3 ±8.7 ^A^	52.6 ±9.3 ^A^	51.1 ±4.9 ^A^	44.5 ±3.9 ^A^
	24	16.7 ±1.5 ^A^	29.9 ±2.8 ^A^	16.8 ±1.6 ^A^	27.4 ±3.7 ^B^	22.4 ±1.2 ^A^	25.7 ±1.2 ^B^	6.3 ±0.8 ^A^	10.3 ±0.9 ^A^
	48	6.9 ±0.6 ^A,^	12.4 ±0.2 ^A,^	5.5±0.2 ^A^	11.7 ±0.3 ^B^	8.7±0.6 ^A^	11.6 ±1.3 ^B^	2.7 ±0.6 ^A^	0.5 ±0.1 ^A^
Path Velocity (VAP, µm/s)	0	168.4 ±17.5^A^	152.0 ±17.5^A^	168.5 ±14.6^A^	152.8 ±12.6^A^	158.7 ±4.2 ^A^	137.1 ±5.1 ^A^	149.8 ±14.5 ^A^	149.6 ±11.7 ^A^
	24	74. ±7.1 ^A,^	111.5 ±23.2 ^B^	75.4 ±11.4 ^A^	108.1 ±12.4^B^	81.4 ±19.8 ^A^	106.3 ±17.8^B^	57.1 ±10.5 ^A^	73.5 ±5.2 ^A^
	48	64.7 ±4.4 ^A^	88.8 ±13.5 ^B^	58.6 ±4.2 ^A^	84.1 ±11.9 ^B^	60.6 ±7.5 ^A^	84.1 ±11.5 ^B^	50.3 ±10.4 ^A^	45.1 ±3.1 ^A^
Progressive velocity (VSL, µm/s)	0	130.2 ±18.3 ^A^	125.4± 7.8 ^A^	138.7 ±12.6 ^A^	125.8±12.8 ^A^	129.5±19.7 ^A^	125.9±12.8 ^A^	126.7±17.1 ^A^	129.3±14.7 ^A^
	24	53.6 ±2.4 ^A^	86.2±2.4 ^B^	52.3 ±11.5 ^A^	73.9±3.7 ^B^	59.1 ±8.3 ^A^	80.4±7.9 ^B^	37.7±7.1 ^A^	54.2±26.6 ^B^
	48	40.5 ±6.3 ^A^	63.6±11.2 ^B^	38.4 ±2.5 ^A^	62.4±8.3 ^B^	38.8 ±6.5 ^A^	60.6±10.3 ^B^	32.7±8.5 ^A^	25.2±4.5 ^A^
Curvilinear velocity (VCL, µm/s)	0	251.5±41.8^A^	252.4±21.3 ^A^	277.5±23.7 ^A^	252.8±12.6^A^	250.4±22.3^A^	244.1±30.5 ^A^	245.4±23.3 ^A^	220.1±27.3^A^
	24	131.7±11.6 ^A^	192.3±48.6 ^B^	132.6±20.5 ^A^	194.5±24.1 ^B^	141.4±14.1 ^A^	191.1±23.3 ^B^	102.2±22.7 ^A^	143.7±27.1 ^B^
	48	110.5±13.2 ^A^	149.6±29.2 ^B^	107.4±6.3 ^A^	158.1±25.4 ^B^	108.8±12.3 ^A^	155.9±21.6 ^B^	84.0±18.2 ^A^	82.2±13.6 ^B^
Lateral head amplitude (ALH, µm)	0	8.8±0.7 ^A^	8.5±0.9 ^A^	9.1±0.9 ^A^	8.5±0.4 ^A^	9.0±0.7 ^A^	8.8±0.8 ^A^	8.3±0.8 ^A^	8.0±0.8 ^A^
	24	6.1±0.6 ^A^	8.5±1.6 ^A^	6.3±0.8 ^A^	8.4±0.8 ^A^	6.3±1.3 ^A^	8.5±0.9 ^A^	4.5±1.6 ^A^	7.2±1.7 ^A^
	48	6.1±0.9 ^A^	7.9±1.3 ^A^	5.7±0.4 ^A^	8.4±1.4 ^A,^	5.3±0.3 ^A^	7.6±0.8 ^A^	3.3±1.9 ^A^	4.0±4.3 ^A^
Beat cross frequency (BCF, Hz)	0	17.7±4.9 ^A^	19.1±4.2 ^A^	17.6±3.3 ^A^	19.2±5.6 ^A^	18.2±5.1 ^A^	18.3±3.9 ^A^	23.6±5.6 ^A^	21.7±2.6 ^A^
	24	14.3±2.7 ^A^	18.1±2.7 ^A^	13.1±2.7 ^A^	18.5±1.5 ^A^	13.7±2.5 ^A^	17.1±3.8 ^A^	12.5±2.8 ^A^	29.5±3.4 ^A^
	48	14.8±1.9 ^A^	16.4±1.6 ^A^	15.1±4.0 ^A^	18.8±3.2 ^A^	14.5±4.9 ^A^	16.3±2.5 ^A^	10.9±5.4 ^A^	17.8±9.1 ^A^
Straightness (STR, %)	0	77.6±4.3 ^A^	77.6±4.3 ^A^	78.6±3.1 ^A^	77.7±2.7 ^A^	77.3±3.1 ^A^	77.7±3.1 ^A^	80.4±4.8 ^A^	83.0±8.0 ^A^
	24	67.1±5.7 ^A^	71.4±2.4 ^A^	64.3±4.6 ^A^	72.7±1.7 ^A^	67.1±5.0 ^A^	70.4±4.7 ^A^	62.9±6.8 ^A^	63.0±8.5 ^A^
	48	62.7±6.1 ^A^	68.1±3.4 ^A^	63.7±5.0 ^A^	69.2±3.1 ^A^	63.7±3.4 ^A^	68.7±3.8 ^A^	64.3±10.4 ^A^	57.5±14.3 ^A^
Linearity (LIN, %)	0	47.1±3.3 ^A^	47.4±4.4 ^A^	48.3±2.3 ^A^	48.3±2.3 ^A^	47.0±1.64 ^A^	47.6±3.6 ^A^	50.4±4.6 ^A^	48.5±6.6 ^A^
	24	40.1±3.9 ^A^	40.9±0.9 ^A^	38.7±2.7 ^A^	38.7±2.7 ^A^	40.6±2.9 ^A^	39.9±2.0 ^A^	40.0±7.1 ^A^	37.0±4.3 ^A^
	48	38.0±3.1 ^A^	39.1±2.3 ^A^	39.0±5.2 ^A^	39.0±5.2 ^A^	38.5±2.6 ^A^	39.3±2.9 ^A^	43.6±8.6 ^A^	35.2±9.9 ^A^

Note: Upper-case superscripts are used for comparisons between the two semen extenders at different storage time for specific EBN concentration. Variables (mean ± SE) with different superscripts indicate significant difference (*P < 0.05*).

For EquiPlus®, PM was not significantly different between control, 0.12% and 0.24% at 24 and 48 h, but 0.24% had higher PM (*P < 0.05*) compared to 0.24% + SP (Figure 2). The PM for E-Z mixin® at different concentrations for each time frame were similar to that of EquiPlus®. Sperm PM for EquiPlus® and E-Z mixin® at each concentration of EBN decreased significantly (*P < 0.05*) from 0 to 48 h. E-Z mixin® resulted to significantly higher PM in 0.12%, compared to EquiPlus® at 24 and 48 h (Table 3).

For EquiPlus®, VAP was not significantly different between control, 0.12% and 0.24%, but 0.24% had higher (*P < 0.05*) VAP compared to 0.24% + SP at 24 and 48 h. VAP decreased significantly from 0 to 48 h following supplementation with 0.12% and 0.24% concentration of EBN. For control and 0.24%+SP, VAP decreased significantly from 0 to 24 h, but there was no difference between 24 and 48 h storage time (Table 2). For E-Z mixin®, VAP decreased significantly from 0 to 48 h for each concentration of EBN (*P < 0.05*). In control, 0.12% and 0.24%, E-Z mixin® performed better at 24 and 48 h as the VAP was significantly higher than that EquiPlus® (Table 3).

For semen extended with EquiPlus®, VSL decreased significantly from 0 to 48 h in control, 0.12% and 0.24%, excluding 0.24% + SP where the values did not differ between 24 and 48 h. For E-Z mixin®, VSL was not significantly different between control, 0.12% and 0.24%, but 0.24% had higher VSL (*P < 0.05*) compared to 0.24% + SP at 24 and 48 h. In addition, VSL decreased significantly from 0 to 48 h in control, 0.12% and 0.24%, and 0.24% + SP (Table 2). Overall, E-Z mixin® performed better at 24 and 48 h as the VSL were significantly higher compared to that of EquiPlus® in control, 0.12% and 0.24% EBN concentrations.

The VCL for EquiPlus® at different concentrations of EBN were not significantly different at 0 h and 24 h, but 0.24% + SP had significantly lower VCL at 48 h compared to 0.24% (Table 2). Likewise, VCL decreased significantly from 0 h to 48 h in all treatments. For E-Z mixin^®^, VCL was not significantly different between control, 0.12% and 0.24% at 0 h and 24 h *(P > 0.05)*, but 0.24% had significantly higher *(P < 0.05)* VCL than 0.24% + SP at 48 h. VCL decreased significantly from 0 h to 48 h for all EBN concentrations *(P < 0.05)*.

In control, 0.12% and 0.24%, E-Z mixin® performed better at 24 h and 48 h as the VCL were significantly higher compared to EquiPlus®. *(P < 0.05)* (Table 2). ALH was not significantly different between the various EBN concentrations in the both extenders. Similarly, supplementation of semen with EBN concentrations had no effect on the LIN, STR and BCF in either the E-Z mixin® or EquiPlus® group (Table 3). However, in 0.24% + SP, E-Z mixin® had significantly higher BCF at 24 and 48h storage time compared to EquiPlus® *(P < 0.05)*.

In terms of the percentage of viable spermatozoa (VAI), for sperm treated with EquiPlus®, no significant difference was observed between the different concentrations of EBN at 0 h. At 24 h, percentage of viable spermatozoa was significantly higher in control (*P < 0.05*) compared to 0.24% + SP. VAI decreased significantly from 0 to 48 h in control and 0.12% (Figure 3). However, VAI decreased significantly (*P < 0.05*) in 0.24% and 0.24% + SP from 0 to 24 h.

For E-Z mixin®, percentage of viable sperm was not significantly different between control, 0.12%, and 0.24%, but the latter had higher value *(P<0.05)* than 0.24% + SP at 0 h. Percentage of viable sperm decreased significantly from 0 to 24 h in control, 0.24% and 0.24% + SP, but no difference was observed between 24 and 48 h. Sperm extended with E-Z mixin® and supplemented with 0.12% EBN concentration had significantly lower (*P < 0.05*) percentage of viable sperm at 48 h compared to other treatments (Figure 3). Comparisons between the two extenders showed that percentage of viable sperm was significantly higher *(P < 0.05*) in E-Z mixin® than EquiPlus® at 0 and 48 h in control and 0.12% EBN concentrations (Table 3).

## Discussion

In this study, we evaluated the effect of two extenders (E-Z mixin® and EquiPlus®) supplemented with different EBN concentrations (control [0.0%], 0.12%, 0.24% and 0.24% + SP) and different storage time (0, 24 and 48 h) on fresh and chilled semen quality of Arabian stallion. Semen diluted with E-Z mixin® at 0.12% EBN concentration had significant higher percentage of sperm total and progressive motility at 24 h and 48 h of storage compared to EquiPlus®. This result is in agreement with other authors who found that different semen extenders induce specific changes in stallion sperm PM (Blanchard et al., 2010; Alamaary et al., 2019). Other authors reported positive correlation between sperm PM and fertility rate after comparing the effects of INRA Freeze® and EquiPlus® (Alamaary et al., 2019). Thus, improvement in PM following supplementation of E-Z mixin® with 0.12% EBN requires further investigation to ascertain its potential to improve fertility rate in mares. There is data paucity on the effects of these two extenders on stallion chilled semen quality. Nevertheless, EquiPlus® extender has been used successfully for liquid storage of stallion semen, but there are indications of its lower efficacy compared to other egg-yolk based extenders in other species (LeFrapper et al., 2010). For instance, in dromedary camel, EquiPlus® extender was ineffective in preserving sperm quality longer than 24 h of storage (Al-Bulushi et al., 2019), while the motility-preserving effect of the extender was only recorded at 24 h (Morton et al., 2013). Although the animals are different, it gives us an insight on varying efficacies between these extenders in preserving semen quality parameters, especially those relating to motility.

Although, this is the first study to assess the positive impact of EBN on stallion sperm quality, there are certain mechanisms that could be responsible for the observed findings. EBN contains carbohydrates and sugar fractions, with previous studies reporting improved motility following supplementation with extenders incorporated with sugars or sucrose-based (Yusop et al., 2016; Consuegra et al., 2018), non-enzymatic anti-oxidants, and sorbitol (Pojprasath et al., 2011). Aside from carbohydrates, EBN contains proteins and trace amount of minerals including sodium and magnesium (Marcone, 2005). Sialic acid is part of EBN’s protein component, and it enhances metabolism and physiological functions in mammals (Yagi et al., 2008), and was reported to maintain sperm morphology during preservation (Yusop et al., 2016).

The improvement in TM and PM in the E-Z mixin® treated semen following the addition of 0.12% of EBN could be related to the anti-oxidant activity of EBN in reducing varying degree of sperm damage during preservation process. Anti-oxidative capacities of EBN has been demonstrated in various experimental studies (Yida et al., 2015; Yusop et al., 2016). EBN attenuated experimentally-induced inflammation and oxidative stress, with high expression of hepatic antioxidant genes associated with better anti-oxidant status (Ismail et al., 2010). Chilled sperm are exposed to high reactive oxygen species (ROS) and reduced anti-oxidant levels, which predispose the spermatozoa to lipid peroxidation (LPO) and apoptosis-like mechanisms leading to premature aging and DNA fragmentation (Aitken and Krausz, 2001; Ortega-Ferrusola et al., 2007; Aitken et al., 2013). Membrane integrity needs to be maintained for the effective permeability and ability of the spermatozoon to regulate the influx of intracellular ions involved in sperm motility (Baumber et al., 2005). The present findings suggest that supplementation of semen extender with EBN concentration, especially at 0.12%, promotes some of these functions in chilled Arabian stallion spermatozoa. Additives with antioxidant properties as exemplified in EBN have been reported to decrease the impact of ROS and cold shock damage (Amidi et al., 2016).

Furthermore, result also showed that supplementation with higher EBN concentration (0.24%) had no significant effect on the total sperm motility. This could be due to impact of the extreme doses of anti-oxidants in the supplement at such concentrations, which may antagonize the oxidative stress as well as disrupting the normal sperm function. Such dose-dependent effects were reported in previous studies (Yida et al., 2015).

Both extenders showed significant decrease in VAP and VSL during the storage time (0, 24 and 48 h) at various EBN concentrations, except for EquiPlus® where no difference was observed in the parameters between 24 and 48 h at 0.24% + SP. These results are consistent with studies reporting significant reduction in sperm VAP and VSL during the storage period using various extenders (Al-Bulushi et al., 2019). Nevertheless, in the groups of control, 0.12% and 0.24 EBN, the VAP and VSL were significantly higher in the E-Z mixin® treated semen compared with the EquiPlus® at 24 and 48 h. Hence, these parameters were greatly improved in the E-Z mixin® group at different concentrations of EBN, except when SP was added. This finding is consistent with studies reporting improved VAP and VSL in post-thawed semen treated with various extenders (Peris et al., 2007; Alamaary et al., 2019). Specifically, the findings agree with those of Waheed et al. (2016), as VAP and VSL were significantly improved by using E-Z mixin® at the dilution rate of 1:1 and 1:2 in preserved horse semen. These parameters are vital in influencing the distance covered by sperm upon entry into the mare’s reproductive tract (Kathiravan et al., 2008). For better fertility rate, sperm needs to cover a long distance at short time intervals in line with the VAP (Kathiravan et al., 2008). Hence, this finding further reinstate the potential of semen treated with E-Z mixin® and supplemented with EBN in inducing better fertility.

Sperms activity may either exhibit positive or negative motility pattern and the parameters to understand these features are VCL and LIN. A sperm extender is judged to be good if it induces high VCL and low LIN values as described by other authors (Oliveira et al., 2013). In this study, VCL was greatly improved in the E-Z mixin® treated semen compared with EquiPlus® for all EBN concentrations at 24 h and 48 h of storage time, except for 0.24% + SP. This outcome could be an indication of better sperm fertilizing ability. Fertility was found to be highly correlated to sperm with high VCL and low LIN values (De Oliveira et al., 2013). Regarding the better performance of E-Z mixin® compared to EquiPlus®, the high concentrations of sugars in E-Z mixin® extender might induce more protection to the sperm capacity (De Oliveira et al., 2013).

In both extenders, the addition of 0.24% + SP resulted in poor sperm motility parameters as the treatment failed to yield significant improvement in PM, TM, VAP, VCL, BCF, and STR. Our findings indicated that supplementation the semen sample with 0.12% and 0.24% concentrations of EBN were effective in preserving the semen motility at various storage time, however, the addition of SP did not maintain the positive impact. Prolonged exposure of sperm to seminal plasma has been reported to induce deleterious effects on sperm kinetics, including the motility and velocity, whereas sperm motility improved after reducing the proportion of SP during cooled storage (Neuhauser et al., 2019). Features such as capacitation and binding to zona pellucida were significantly reduced in sperm cells exposed to SP suggested to be due to the decapacitating effect of intrinsic SP proteins (Al-Essawe et al., 2018b). Other factors influencing post-thaw sperm quality as related to SP include the exposure time, origin and composition, semen fraction, season and stallion’s age (Al-Essawe et al., 2018b). In the present study, it could be that the underlining mechanisms through which EBN augments extenders in preserving sperm quality lags behind the deleterious effects of SP in chilled semen. Nevertheless, we need to consider that only 0.24% + SP was included in the experimental groups, and the same event might not be applicable to 0.12% EBN concentration.

Viability test is used in assessing the modifications of sperm plasma membrane and sperm morphology (Al-Essawe et al., 2018a). In the present study, E-Z mixin® had higher % viable in control samples compared to EquiPlus®. Although the values decreased at increasing storage time, E-Z mixin® was still able to achieve higher % viable at 48 h. As found in this study, decrease in the % viable of spermatozoa diluted in commercial extenders, following liquid storage and storage time (0-48 h) has been reported using camel semen (Al-Bulushi et al. 2019). Further, comparisons between the two extenders showed that % viable was significantly higher in E-Z mixin® than EquiPlus® at 0 and 48 h in control and 0.12% EBN concentration. This result highlights that E-Z mixin® was able to preserve higher proportion of viable spermatozoa at higher concentrations of EBN compared to EquiPlus®. Studies have shown that different extenders when supplemented with certain agents may exert varying effects on percentage viable spermatozoa in horses (De Oliveira et al., 2013; Al-Kass et al., 2018). The acrosome membrane may be rendered non-intact by local production of ROS (reactive oxygen species) (Whittington and Ford, 1999), which ultimately lead to oxidative stress characterized with marked reduction in viability, increased DNA damage, and morphological abnormalities (Aitken et al., 2013). The application of extenders to reduce this effect may be influenced by their anti-oxidant properties (Usuga et al., 2017).

## Conclusion

The results of this study indicated that chilled semen from Arabian stallion that was extended using E-Z mixin® and supplemented with 0.12% and 0.24% EBN concentrations performed better and yielded superior results in sperm kinetic parameters compared to EquiPlus® at 24 and 48 h storage time. Supplementation of the E-Z mixin® extender with 0.12% and 0.24% EBN concentrations in the absence of SP provided better CASA parameters such as TM, PM, VAP, VSL and VCL at 24 and 48 h storage times. Further research is needed to elucidate the mechanisms underlining the positive effects of EBN supplemented extenders on sperm kinetics in Arabian stallion semen.
